# Associations of Eating Mode Defined by Dietary Patterns with Cardiometabolic Risk Factors in the Malaysia Lipid Study Population

**DOI:** 10.3390/nu12072080

**Published:** 2020-07-14

**Authors:** Gaiyal Viliy Balasubramanian, Khun-Aik Chuah, Ban-Hock Khor, Ayesha Sualeheen, Zu-Wei Yeak, Karuthan Chinna, Kalyana Sundram, Tilakavati Karupaiah

**Affiliations:** 1 Dietetics Program, Faculty of Health Sciences, Universiti Kebangsaan Malaysia, Jalan Raja Muda Abdul Aziz, Kuala Lumpur 50300, Malaysia; gaiyalviliy@gmail.com (G.V.B.); aishaltaf@ymail.com (A.S.); 2 Nutrition Program, Faculty of Health Sciences, Universiti Kebangsaan Malaysia, Jalan Raja Muda Abdul Aziz, Kuala Lumpur 50300, Malaysia; cruise_chuah@hotmail.com (K.-A.C.); yeak_wei@hotmail.com (Z.-W.Y.); 3 Department of Medicine, Faculty of Medicine, Universiti Kebangsaan Malaysia, Bandar Tun Razak, Kuala Lumpur 56000, Malaysia; khorbanhock@gmail.com; 4 School of Medicine, Faculty of Health and Medical Sciences, Taylor’s University, Subang Jaya, Selangor 47500, Malaysia; karuthan@gmail.com; 5 Malaysian Palm Oil Council, Menara Axis, Petaling Jaya, Selangor 46100, Malaysia; kalyana@mpoc.org.my; 6 School of BioSciences, Faculty of Health and Medical Sciences, Taylor’s University, Subang Jaya, Selangor 47500, Malaysia

**Keywords:** dietary pattern, sugar-sweetened beverages, home meal, atherogenicity, metabolic syndrome

## Abstract

Cardiometabolic risk is scarcely explored related to dietary patterns (DPs) in Asian populations. Dietary data (*n* = 562) from the cross-sectional Malaysia Lipid Study were used to derive DPs through principal component analysis. Associations of DPs were examined with metabolic syndrome (MetS), atherogenic, inflammation and insulinemic status. Four DPs with distinctive eating modes were Home meal (HM), Chinese traditional (CT), Plant foods (PF) and Sugar-sweetened beverages (SSB). Within DP tertiles (T3 vs. T1), the significantly lowest risk was associated with CT for hsCRP (AOR = 0.44, 95% CI 0.28, 0.70, *p* < 0.001) levels. However, SSB was associated with the significantly highest risks for BMI (AOR = 2.01, 95% CI 1.28, 3.17, *p* = 0.003), waist circumference (AOR = 1.81, 95% CI 1.14, 2.87, *p* = 0.013), small LDL-C particles (AOR= 1.69, 95% CI 1.02, 2.79, *p* = 0.043), HOMA2-IR (AOR = 2.63, 95% CI 1.25, 5.57, *p* = 0.011), hsCRP (AOR = 2.21, 95% CI 1.40, 3.50, *p* = 0.001), and MetS (AOR = 2.78, 95% CI 1.49, 5.22, *p* = 0.001). Adherence behaviors to SSBs (T3) included consuming coffee/tea with condensed milk (29%) or plain with sugar (20.7%) and eating out (12 ± 8 times/week, *p* < 0.001). Overall, the SSB pattern with a highest frequency of eating out was detrimentally associated with cardiometabolic risks.

## 1. Introduction

Dietary risks contribute substantively to the disability-adjusted life-years (DALYs) burden from non-communicable diseases (NCDs) in the Global Burden of Disease ranking [1], and this risk is prominent in the South East Asian Region (SEAR), irrespective of economic disparities [2]. Percent change for dietary risks from 2007 to 2017 rose by 29.2% for Malaysia, which carries the highest DALY ranking within the SEAR, [1]. Transitional change to unhealthy diets along with lifestyle changes are implicit in population-wide behavior risks.

Evidence links from diets to disease traditionally address single nutrient–calorie approaches, but the last decade has seen a shift to ‘single food’ and ‘whole food’ approaches, with dietary guidelines promoting desirable diets for populations [3,4,5,6]. The transferability of whole diets, such as the Mediterranean diet designed for the Western template, challenges application to the SEAR countries [7,8], as dietary habits in this region are heterogeneous and driven by cultural diversities of race, religion and food availability. Contrarily, a ‘meal centric’ approach enables the identification of dietary patterns within local populations that reveal food combinations within dishes and meals, as well as the ‘eating mode’. This is an urgent research agenda in population nutrition, in order to build evidence for food-based dietary guidelines.

Dietary patterns define food evolution in the context of local food availability, culture and ethnic practices, and may relate to obesity and NCDs [9]. Asian studies in Singapore [10], Thailand [11], Bangladesh [12] and Middle Eastern countries such as Qatar [13] and Lebanon [14] highlighted unhealthy dietary patterns, concentrating on animal proteins, fast foods with sugar and carbohydrates, as being associated with NCD risk. A meta-analysis of 40 global studies [15] showed a “Healthy” dietary pattern associated with an overall 15% reduction in metabolic syndrome (MetS) risk in East Asian countries, whilst adherence to the “Meat/Western” type pattern increased MetS risk by 19% across Asia, Europe or America. The meta-analysis included subjects diagnosed with medical history of diabetes or hypertension and only reported outcomes related to MetS criteria. None of these studies included populations without medical history of NCDs. Furthermore, none included comprehensive cardiometabolic profiles of inflammation status, atherosclerotic vascularization and insulin resistance, which are early signals in the pathways of MetS risk.

For the first time, Malaysian schoolteachers were examined for “Western” and “Prudent” dietary pattern practices, but the study in question did not explore associations with disease risk, nor did ethnic driven food patterns emerge from this analysis [16]. Recently, our group published findings from the Malaysia Lipid Study (MLS) showing atherogenic and cardiometabolic burden of high carbohydrate-high fat diets [17] in an urban-living cohort. The primary approach to the MLS data analyses was to report on macronutrient proportions congruent with cardiometabolic health risks but did not identify foods that carried this risk. Our intention now for this secondary analysis of the MLS data was to define the diet–food matrix of this urban Malaysian population in terms of dietary patterns, which will serve to elucidate the ‘eating mode’ as determined by lifestyle, cultural diversity, age and gender. The purpose of this study, therefore, was to examine the associations between à posteriori-derived dietary patterns of MLS subjects with risk of MetS and cardiometabolic profiles relating to atherogenic, inflammation and insulinemic status. This would inform future prevention strategies to drive health promotion inclusive of a locally specific personal medicine approach.

## 2. Materials and Methods

### 2.1. Study Population

The population setting was provided by the MLS [17]. MLS was conducted between November 2012 and November 2013, with the aim of recruiting a typical palm oil consuming adult population living in an urban setting. The methods used for MLS cohort formation and its baseline characteristics relating to macronutrient composition and cardiometabolic risk have been described elsewhere [17]. Essentially, this study population was multiracial in composition. The selection criteria for MLS was a chronic disease diagnosis-free population without any smoking history. In this 2nd cycle of MLS analysis, 15 participants were excluded due to missing dietary history records, leaving food records for 562 participants. The study protocol was approved by the institutional medical review board of Universiti Kebangsaan Malaysia (UKM 1.5.3.5/138/NN-047-2012). All participants provided written informed consent.

### 2.2. Dietary Data Consolidation

Three days of dietary records (3DDRs) randomized per subject for two weekdays and one weekend-day formed the database along with a dietary history capturing information on lifestyle patterns. The collection of dietary data followed the 24 h dietary recall methodology cited by the National Health and Nutrition Examination Survey [18]. Subjects were provided with standard household units to increase the recall accuracy of their dietary records. Dietitians who collected these records verified them through interviews with subjects. Interviewers were provided training for standardization of interview techniques so as to minimize inter-measurement error [19].

Food items reported in the 3DDRs were transformed into gram units based on actual food weights recorded in our laboratory. Nutrient intake data were analyzed using Nutritionist-Pro∑ software (First-databank Inc., Chicago, IL, USA), which includes the Malaysian, Singaporean and United States Department of Agriculture Food Composition databases [20]. For cooked foods that were not available in these databases, the standard recipe was constructed using Nutritionist Pro∑ software [21]. Food labels were referred for processed foods. Where the food item’s recipe or food packaging were unavailable, the nutrient content of a similar food was substituted.

### 2.3. Food Grouping

Food items relating to meals and beverages extracted from 3DDRs were alphabetically re-arranged, duplicates removed, and the final listing was grouped according to similarity, culinary use and nutrient content. Initially, 40 food groups were formed. A dietary pattern template was then created for each participant to integrate the food group listing. This template enabled data imported from Nutritionist-Pro∑ to be assigned to the food groups. Mean consumption for each food group per subject per day was then computed. Food items consumed by less than 5% of participants were excluded, which narrowed the final food listing to 23 food groups, with foods within categories sharing a similar nutritional profile.

### 2.4. Demographic Characteristics

Anthropometry, biochemistry and clinical data from MLS subjects were integrated with dietary patterns defined through principal component analysis (PCA). The parameters were body mass index (BMI), waist circumference (WC), lipid profile, plasma glucose and insulin, calculated homeostasis model assessment of insulin resistance (HOMA2-IR), high sensitivity C-reactive protein (hsCRP), systolic (SBP) and diastolic (DBP) blood pressure and lipoprotein particle size. The details of these measurements were reported in the primary paper [17]. These data allowed the computing of a range of cardiometabolic markers including the criteria for MetS diagnosis [22].

### 2.5. Sociodemographic, Income and Lifestyle Variables

The data collected from MLS included ethnicity, household income, occupational status, dietary history, which included eating out practices, and physical activity level.

### 2.6. Statistical Analysis

Dietary patterns using the à posteriori approach were derived using PCA of mean consumption data of subjects, based on the correlation matrix of the 23 food groups. PCA was performed using Statistical Package for Social Sciences, SPSS^®^ for Windows™ application version 23.0 (IBM, Chicago, IL, USA). Factors were rotated by the varimax method to improve the interpretability of factors. Key dietary patterns were identified by (i) selecting factors with eigenvalues >1, (ii) analyzing the scree plot to retain the factors in the steep curve before the first point that starts the flat line, and (iii) factor interpretability [9,23]. The patterns that emerged were named according to food groups that carried the maximum factor loadings >|0.30| for each pattern. Every subject was assigned a factor score for each dietary pattern related to food items in food groups identified through their 3DDR data. The factor scores were then categorized into tertiles for each dietary pattern.

Descriptive data relating to demographic, social, income and lifestyle characteristics of the final number of subjects were compared against tertiles (T1 to T3) of dietary patterns using the chi-square tests for categorical variables and ANOVA for continuous variables. Data on food group consumptions were presented as medians (interquartile range), and the Kruskal–Wallis test was used for comparison against tertiles (T1 to T3) of dietary patterns. The general linear model procedure was used to compare cardiometabolic risk markers between tertiles. Three models were reported: Model 1, unadjusted, Model 2, adjusted for age and gender, and Model 3, adjusted for age, gender, education level, income, and physical activity level. Logistic regression analysis, adjusted for age, gender, education level, income, and physical activity level, was used to determine risk associations between tertile extremes (T3 vs. T1) within identified dietary patterns with criteria for MetS cutoffs (individual and diagnosis) and elevated cardiometabolic outcomes relating to atherogenicity (lipid profile and lipoprotein particle size), inflammation (hsCRP) and insulinemic status (HOMA2-IR). The cut-off values identified for HOMA2-IR index had an epidemiological application for identifying MetS in Westernized multi-ethnic populations as recommended by Geloneze et al. [24]. Two-sided *p* values < 0.05 were considered statistically significant. These statistical analyses were also carried out using the SPSS^®^ for Windows™ application version 23.0 (IBM, Chicago, IL, USA).

## 3. Results

### 3.1. Characteristics of Study Population

Table 1 provides the demographic characteristics of 562 subjects included in the final analyses, with an ethnic breakdown of 222 (40%) Malays, 201 (36%) Chinese and 139 (25%) Indians. The majority (63%) were females and the majority (60%) had obtained tertiary education. Only 152 (27%) subjects reported being physically active. The mean age was 38.1 ± 11.4 years, and the overall monthly household income was RM 4434 ± 3476.

### 3.2. Dietary Patterns

**Figure 1:**
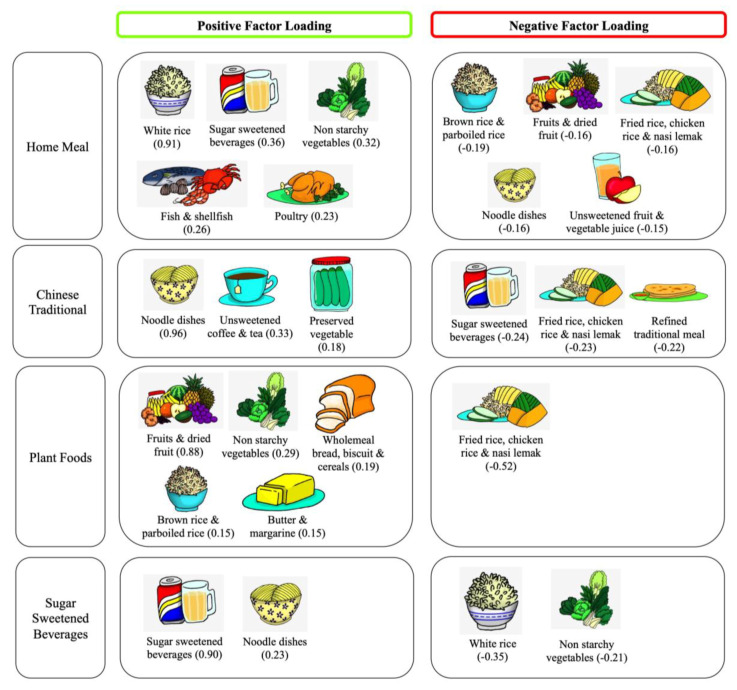
Factor loadings for four dietary patterns in the MLS population (*n* = 562). Dietary pattern extraction is by principal component analysis; numbers in parenthesis indicate factor loading for that food item.

The Kaiser–Meyer–Olkin value of the factor analysis was 0.537. Four dietary patterns with a total of 23 food groups were extracted using PCA (Table 2 and Figure 1). The first factor was labeled as the ‘Home Meal’ (HM) pattern, as it represented a high intake of white rice, sugar sweetened beverages and non-starchy vegetables. The second pattern was labeled as the ‘Chinese Traditional’ pattern (CT), with its high intake of noodle dishes, unsweetened plain coffee and tea. The third pattern reflecting a high intake of fruit and non-starchy vegetables, was labeled as the ‘Plant Foods’ pattern (PF). The last pattern was labeled as the ‘Sugar-Sweetened Beverages’ pattern (SSB), as it represented a high intake of sugar-sweetened beverages. All four patterns had eigenvalues more than 1.0 with cumulative percentage variance of 76.3%, with the SSB pattern accounting for the highest variation (35.1%), followed by the CT (20.3%), HM (13.3%) and PF (7.6%) patterns.

### 3.3. Dietary Pattern Comparisons by Tertiles of Population Characteristics

Associations between demographic characteristics, nutrient intake, eating out frequency and income are presented in Table 3. In terms of age, subjects in the highest tertile (T3) of consumption were older compared to T1 and T2 subjects in HM (*p* = 0.014) and PF (*p* = 0.002) patterns. Relating to proportion by gender, males by number significantly decreased across tertiles of PF pattern (*P_trend_* = 0.005), but increased across tertiles of SSB pattern (*P_trend_* < 0.001); and these trends reversed in females (*P_trend_* < 0.05).

In terms of nutrient intake, the highest carbohydrate intakes were associated with the highest tertiles (T3) of HM and SSB (both *P_trend_* < 0.001) patterns. T3 levels of protein intake were highest for CT (67.6 ± 18.4 g, *P_trend_* = 0.001) > SSB (67.1 ± 19.6 g, *P_trend_* < 0.001) > HM (67.0 ± 20.9 g, *P_trend_* = 0.007) > PF (63.7 ± 19.5 g, *P_trend_* > 0.05) patterns, whilst T3 levels of fat intake were highest for SSB (*P_trend_* = 0.002) and CT (*P_trend_* = 0.003). Notably T3 subjects of SSB compared to the other dietary patterns were associated with the highest consumption of calories (1964 ± 426 kcal, *p* < 0.001), carbohydrates (270.7 ± 60.8 g, *p* < 0.001) and fat (67.8 ± 20.3 g, *p* < 0.007) intakes. Dietary sodium intake of T3 subjects was the highest for CT (3409 ± 1115 mg, *P_trend_* < 0.001) greater than SSB (3043 ± 984 mg), PF (3013 ± 966 mg), and HM (3010 ± 1019 mg). The PF pattern was similar across all tertiles of energy and macronutrient intakes (all *p* > 0.05). 

The assessment of daily intakes as per food groups consumed by a subject in each tertile was also performed. Accordingly, the T3 subjects of the HM pattern had greater intakes of white rice (*P_trend_* < 0.001), non-starchy vegetables (*P_trend_* < 0.001), fish and shellfish (*P_trend_* < 0.001), and poultry (*P_trend_* < 0.001), with lower consumption of noodle dishes (*P_trend_* < 0.001). Contrarily, the T3 subjects of the CT pattern had the greatest intake of noodle dishes (*P_trend_* < 0.001), but the lowest consumptions of white rice (*P_trend_* = 0.002), legume (*P_trend_* = 0.010), fried rice and *nasi lemak* (*P_trend_* = 0.016), and refined traditional cereal (*P_trend_* = 0.002). T3 subjects of PF pattern had greatest consumption of fruit and dried fruit (*P_trend_* < 0.001). With the SSB pattern, T3 subjects had the highest intakes of egg (*P_trend_* = 0.038), *kuih* (*P_trend_* = 0.005), fried rice and *nasi lemak* (*P_trend_* = 0.050), refined traditional cereal (*P_trend_* < 0.001), and sugary beverages (*P_trend_* < 0.001), but the lowest consumption of non-starchy vegetables (*P_trend_* = 0.009) as well as fruit and dried fruit (*P_trend_* = 0.001).

In terms of ethnicity, the HM pattern was similarly followed by proportions of Malays, Chinese and Indians across tertile comparisons (all *P_trend_* > 0.05). The CT pattern was significantly dominated by Chinese subjects (*P_trend_* < 0.001), as indicated by 57% in T3 and 21% in T2. In the PF pattern, there were more Malays in T1 (44%) but more Chinese (47%) in T3 (*P_trend_* = 0.001). In the SSB pattern, there were more Chinese (47%) in T1 but more Malays (46%) in T3 (*P_trend_* = 0.001). Comparatively, Indians displayed no clear choice as per T3 comparisons (*P_trend_* > 0.05) across HM (24%), PF (20%) and SSB (28%) patterns, but chose CT the least (8%).

The frequency of eating out across T3 comparisons indicated the highest weekly frequency was associated with the CT (11 ± 7 times/week, *P_trend_* = 0.006) and SSB (12 ± 8 times/week, *P_trend_* < 0.001) patterns, whereas the lowest frequency was associated with the PF pattern (9 ± 7 times/week, *P_trend_* = 0.047). There was no significant difference in incomes across tertiles (all *P_trend_* > 0.05) within or between the four dietary patterns.

### 3.4. Associations between Dietary Patterns and Cardiometabolic Risk Factors

The associations between the four dietary patterns and cardiometabolic risk factors by tertiles are presented in Table 4. Using adjusted co-variate analyses (Model 3), linearly increasing significant trends in BMI (T1 = 24.2 ± 0.4 < T2 = 25.2 ± 0.4 < T3 = 25.7 ± 0.4 cm, *P_trend_* = 0.012) and waist circumference (T1 = 83.5 ± 1.0 < T2 = 84.9 ± 1.0 < T3 = 86.8 ± 1.0 cm, *P_trend_* = 0.038) were associated with the SSB pattern, whereas for HM, higher BMI (*P_trend_* = 0.012) and waist circumference (*P_trend_* = 0.016) were associated only with T2 subjects. Contrarily, the CT pattern was associated with a decreasing waist circumference trend with increasing tertiles (T1 = 86.5 ± 1.0 > T2 = 85.9 ± 1.0> T3 = 83.2 ± 1.0 cm, *P_trend_* = 0.021).

In terms of lipid profile a positive association between SSB and TC:HDL-C was observed (*P_trend_* = 0.039). Limited interactions between T1 and T2 were noted for increased small low-density lipoprotein particles (*P_trend_* = 0.028) and decreased large HDL particles (*P_trend_* = 0.036). Of note, this SSB pattern was detrimental with insulinemic status (plasma insulin, *P_trend_* = 0.022) and HOMA2-IR (*P_trend_* = 0.023).

In contrast, a reversed trend for hsCRP levels associated with CT tertiles (*P_trend_* = 0.043). An opposite trend (*P_trend_* = 0.015) as regards inflammatory status was observed with the SSB pattern (*P_trend_* = 0.035), although the interaction was limited between T1 and T2.

As regards the PF pattern, between-tertile differences were not significant for all variables (all *P_trend_* > 0.05).

### 3.5. Associations between Dietary Patterns and MetS and Cardiometabolic Risk Factors

**Figure 2:**
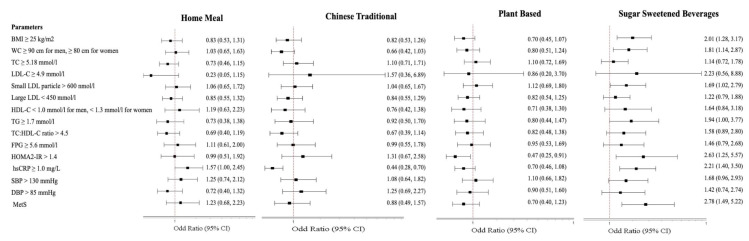
Associations between cardiometabolic markers and T3 vs. T1 of dietary patterns. Data are expressed as odds ratio (95% confidence interval) of T3 against odd ratio of T1 set as 1.0; ^†^ Multiple logistic regression test for comparison between tertiles (T3 vs. T1) adjusted for age and gender. Abbreviations: AOR = adjusted odds ratio; BMI, body mass index; CI, confidence interval; DBP, diastolic blood pressure; FPG, fasting plasma glucose; HDL, high density lipoprotein; HOMA2-IR, homeostatic model assessment of insulin resistance; hsCRP, high-sensitivity C-reactive protein; LDL-C, low density lipoprotein; MetS, Metabolic Syndrome; SBP, systolic blood pressure; TC, total cholesterol; TC:HDL-C, total cholesterol high density lipoprotein ratio; TG, triglyceride; WC, waist circumference.

Multivariate logistic regression analysis was performed with the highest level of consumption for each dietary pattern (T3) against the lowest consumption (T1) as a reference for risk related to cardiometabolic abnormalities and MetS, corrected for co-variates. Data are reported as adjusted odds ratios (AOR) and 95% confidence interval (95% CI) in Figure 2. Detailed data of parameter AOR (95% CI) are included in Supplemental Table S1.

With the HM pattern, the difference was observed as regards hsCRP levels with odds for increased risk in T3 compared to T1 (AOR = 1.57, 95% CI 1.00, 2.45, *p* = 0.049). With the CT pattern, the difference concerned only hsCRP levels, where the odds of high hsCRP risk was lowest with T3 compared to T1 (AOR = 0.44, 95% CI 0.28, 0.70, *p* < 0.001). With the PF pattern, odds for a significantly reduced risk were related to HOMA2-IR (AOR = 0.47, 95% CI 0.25, 0.91, *p* = 0.025).

With the SSB pattern, significant differences related to BMI, waist circumference, small LDL-C, HOMA2-IR, hsCRP and MetS. The odds of high values for all these six variables were significantly higher with T3 compared to T1: BMI (AOR = 2.01, 95% CI 1.28, 3.17, *p* = 0.003), waist circumference (AOR = 1.81, 95% CI 1.14, 2.87, *p* = 0.013), small LDL-C particles (AOR = 1.69, 95% CI 1.02, 2.79, *p* = 0.043), HOMA2-IR (AOR = 2.63, 95% CI 1.25, 5.57, *p* = 0.011), hsCRP (AOR = 2.21, 95% CI 1.40, 3.50, *p* = 0.001), and MetS (AOR = 2.78, 95% CI 1.49, 5.22, *p* = 0.001).

**Figure 3:**
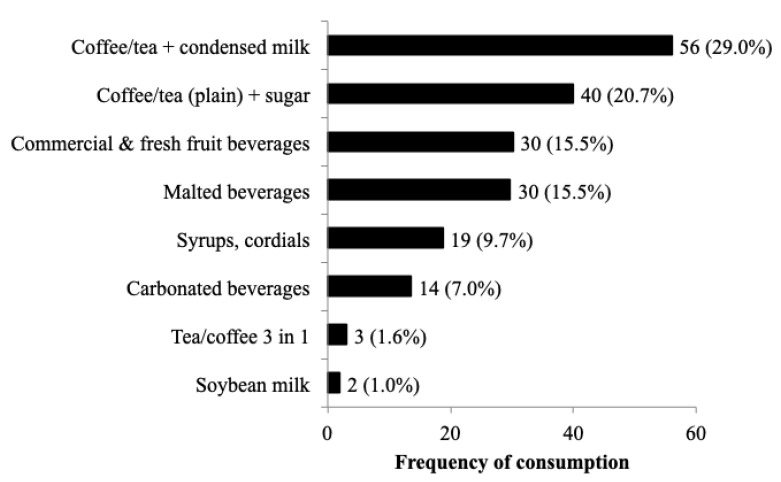
Percent distribution of sugar sweetened beverage choices for SSB T3 subjects (*n* = 193). Footnote: data provided as *n* (%).

Figure 3 provides the reported non-alcoholic beverage consumption breakdown by T3 subjects within the SSB dietary pattern. Tea or coffee were mostly consumed with condensed milk (29%) or plain with sugar (20.7%), followed by commercial/fresh fruit drinks (15.6%), cocoa and malted beverages (15.4%), syrup/cordials (9.7%), and carbonated beverages (7.0%). Soybean-based drinks (1%) and 3-in-1 tea/coffee (1.6%) were minor choices.

## 4. Discussion

This study’s findings are highly relevant to SEAR countries facing the high DALY burden from NCDs attributed to rapid urbanization, increased household incomes, and greater dependence on processed food or eating out. For Malaysia, this is the first time a dietary pattern analysis has been performed for an urban adult population with biomarkers of cardiometabolic, inflammation and insulinemic status [17]. Identifying dietary patterns within this population revealed current local exposure to food consumption behaviors. The recruited population represented young to middle-age, middle income adults from three major ethnic groups and essentially free from diagnosis of NCDs. This sampled population effectively reflected the sociodemographic backdrop of Malaysia, against which emerges the high mortality rates attributed to NCDs [25,26]. This sampling is justifiable as Malaysia faces a rapid urban-centric development, with 72.8% of its population (total population: 29,240,000) living in urban areas, and with a growing middle class [27,28]. Its heterogeneous population is drawn from three main ethnic groups—Malay, Chinese and Indian, together making up 95% of the total population [29].

The four identified dietary patterns in this study together accounted for 76.3% of total variance, with the SSB pattern (35.1%) contributing the largest proportion. We observed distinctive ethnic-centric CT, PF and SSB patterns influenced by culture and lifestyle, which differentially associated with cardiometabolic biomarkers related to NCDs. The SSB pattern was dominant with Malay and Indian subjects, whilst the CT and PF patterns were dominant with Chinese subjects. Men were least likely to consume the low-risk plant-based PF pattern but most likely to consume the unhealthy SSB pattern, whereas these trends reversed in women. Gender-specific dietary pattern choices have also been observed in China, with men favoring the “animal and fried food” pattern and women favoring the “high-salt and energy” pattern [30], or in Korea, where men favored a “meat pattern” [31]. Despite a systematic review noting that socioeconomic status was a determinant of dietary patterns in low- and middle-income countries [32], income did not influence pattern adherence in this Malaysian population. Instead, increased eating out practices of T3 subjects were associated with both CT and SSB patterns, but only cardiometabolic risk potential was associated with SSB. This is attributed to high sweetened beverage consumption in SSB subjects compared to a preference for unsweetened beverage consumption prevailing amongst CT subjects. Older subjects in our study were more likely to consume the HM and PF patterns. 

The main finding from this study was that the SSB pattern alone was contributive to MetS risk in this population, with odds risk at 2.78 times with the highest level of SSB consumption (T3) compared to T1 subjects. These findings concur with a study from Kuwait [33] reporting odds of 2.66 times for MetS associated with a fast-food dietary pattern descriptive of burgers/sandwiches, French fries, and SSBs. However, other Asian findings differently associate MetS risk as being low with a Westernized-breakfast pattern symbolic of bread, confectionary and dairy products in Japanese municipal employees [34] or a traditional pattern carrying a 28% lower risk, whereas the animal food pattern bore a 28% greater risk in China [35]. The perception of increased MetS risk universally associated with the “Meat/Western” dietary pattern as reported in a meta-analysis [15] was clearly not observed in our study. We contend that within-country studies in Asia yield discriminating dietary patterns with cardiometabolic health implications that are unique to a country’s population eating mode.

Relevant to obesity risk, highly adherent SSB pattern behaviors (T3 vs. T1) characterized by non-alcoholic beverages are associated with a doubly higher risk for BMI ≥ 25 kg m^2^ and 81% higher risk for increased waist circumference. Specifically, a large Polish cross-sectional study (*n* = 7997 adults) noted adherence to the “traditional-carbohydrate” pattern, characterized by higher intakes of refined grains, potatoes, sugar and sweets was associated with a higher risk of abdominal obesity [36]. In Kuwait, the fast-food dietary pattern inclusive of burgers/sandwiches, French fries, and SSBs was positively associated with increased BMI and waist circumference [33]. In context, a systematic review and meta-analysis of à posteriori-derived dietary patterns evaluating only central obesity as a single outcome associated a significantly decreased risk with ‘healthy/prudent’ patterns but a non-significantly increased risk with ‘unhealthy/Westernized’ patterns [37].

However, beyond establishing the high MetS risk associated with the SSB pattern, this dietary pattern also associated with greater risk for insulinemic status with linear increases in plasma insulin and HOMA2-IR (both *p* < 0.05) and a non-significant (*p* = 0.09) increased trend in fasting blood glucose. The odds for insulin resistance more than doubled (2.63 times) with SSB contrasting with the 53% reduction associated with the plant-based PF patterns. Additionally, the atherogenic burden of the SSB pattern was pointed out by a linear increase in TC:HDL-C ratio and TG levels across tertiles. An increase in pro-atherogenic small dense LDL lipoprotein particles was denoted by a limited interaction between T1 and T2. This was reinforced by a significant 69% increased odds for the occurrence of small dense LDL particles associated with the SSB pattern. Using a novel approach in the primary macronutrient-centric analyses of MLS data [18], we classified low fat as <50 g, high fat as >70 g, low carbohydrate as <210 g and high carbohydrate as >285 g, yielding four permutations, namely low fat-low carbohydrate, low fat-high carbohydrate, high fat-low carbohydrate and high fat-high carbohydrate. It appears the high fat-high carbohydrate dietary consumption attributing to the atherogenic and insulinemic risks in the primary analyses closely defined the SSB pattern’s associations in this secondary analysis. The cogent explanation for this may be inferred from SSB’s high factor loading (0.90) for beverages with refined sugars contrasting with the HM (0.33) pattern or negative loadings for the CT (−0.24) and PF (−0.05) patterns. Comparatively, rice and noodle consumption in a Singapore population was associated with insulin resistance and hyperglycemia [38].

Do dietary choices pose inflammation risk in disease free populations? This aspect is noted in the literature and the more sensitive hsCRP is a biomarker we explored as a surrogate to address the identification of low-grade inflammation [39]. In line with this, significant hsCRP trends were marked by a linear increase with SSB tertiles and a reversed trend with the CT pattern. This translated into odds for inflammation risk more than doubling in T3 of the SSB pattern (2.21 times), whilst this risk reduced by 56% with the CT pattern. A marked difference between the SSB and CT patterns was although both patterns associated with eating out, the CT pattern carried a negative loading (−0.24) for SSB consumption. A Taiwanese study (*n* = 26,016), using the less sensitive C-reactive protein as an inflammation marker, reported a high intake of a meat-instant food dietary pattern was positively associated with components of MetS and CRP, but high intake of either vegetable–seafood or cereal–dairy indicated inverse effects [40].

At the highest level of consumption (T3), both SSB and HM patterns carried similar high fat-high carbohydrate profiles with the highest calories (1964 ± 426 vs. 1907 ± 453 kcal), carbohydrates (270.7 ± 60.8 vs. 263.3 ± 62.1 g) and fat (67.8 ± 20.3 vs. 64.7 ± 21.5 g) respectively, compared to the other dietary patterns. With only a 57% increased odds risk for high hsCRP levels but otherwise neutral towards the cardiometabolic risk markers studied, HM’s core differences compared to SSB is attributed to having a lower amount for consumption of sugary beverages (342 vs. 605 mL), higher consumption of non-starchy vegetables (82 vs. 47 g) and a substitution with white rice for sugary beverages (281 vs. 143 g). The nature of SSB pattern’s carbohydrate load was characterized by sweetened drinks containing condensed milk and maltose-rich beverages which supported its cardiometabolic risk profile associated with the biomarkers of atherogenicity, insulinemia and inflammation status. In the Singapore Chinese Health Study cohort, total carbohydrate intake could not be associated with IHD mortality risk, despite a high carbohydrate consumption [10]. Instead, refined carbohydrates and low fiber intake posed a risk for IHD death; the replacement of one daily serving of rice with noodles was associated with higher risk, whilst replacing one daily serving of rice with vegetables, fruit, or whole-wheat bread lowered risk.

There is an increasing pattern of literature reporting on the morbidity and mortality risks associated with SSB consumption in large population studies. An the SSB pattern has been linked to a 56% higher hazard ratio from acute coronary heart disease in the Southern Dietary Pattern characterized by added fats, fried food, eggs, organ and processed meats, and sugar-sweetened beverages in the Reasons for Geographic and Racial Differences in Stroke [REGARDS] study [41]. Secondary analyses from REGARDS showed that the consumption of sugary beverages, including fruit juices, was associated with all-cause mortality, although no significant association of sugary beverage consumption was shown with coronary heart disease mortality [42]. A recent combined analysis from both the Health Professional’s Follow-up and the Nurses’ Health indicated that the consumption of SSBs was positively associated with mortality, primarily through cardiovascular mortality and a graded association with dose was shown [43]. However, other prospective studies such as the Singapore Chinese Health Study cohort [44], Adventist Health Study-2 in North America [45] and even the rural-based Bangladesh study [12] indicated that animal protein-rich diets were positively associated with cardiovascular events and mortality. A reverse association with plant-based diets has also been noted [45,46].

The primary strength of this study, lacking in other food-based studies, indicates that this was the first time dietary patterns were elucidated against the wider scope of cardiometabolic risk in terms of lipoprotein particles, hsCRP and HOMA2-IR scores in a disease-free population. A strength in this study is the use of 3-day dietary records which generally overcomes the limitations associated with the use of semi-quantitative food frequency questionnaires in dietary pattern analyses [8]. The use of FFQs assume food behaviors are homogenous to ‘healthy’, ‘prudent’ or ‘meat/Western’ patterns, irrespective of geographical regions, as with the CARRS study comparing cities in India and Pakistan used in the INTERHEART study [46]. Such analyses miss out culturally specific behaviors of population groups within a country, as shown in our study.

There are some limitations to this study. Firstly, the interpretation of data from this study must take into account the cross-sectional nature of the study design, which means that dietary exposures within this population were therefore temporal. There are also limitations inherent with the à posteriori-derived dietary patterns approach such as subjective interpretation of numerous combinations of food and beverage in diets as food items best characterizing the food pattern. However, the à posteriori approach allowed for untargeted analyses based on 24 h dietary recalls in our study compared to the à priori approach or use of semi-quantitative or quantitative food frequency questionnaires.

Discerning discriminating dietary patterns within this population revealed behavior traits linked to ‘eating modes’ that should be targeted in the development of dietary guidelines and public nutrition health messages. The plant-based PF pattern with low eating out frequency bore no risk to any of the examined biomarkers of atherogenicity and inflammation, with an additional advantage of a 53% lower risk of insulin resistance. A 57% increased odds risk for high hsCRP levels associated with HM contrasting with a 56% reduction with the CT pattern. It seemed that neither CT, PF or HM patterns were associated with MetS risk. The different health risk traits of CT, PF and HM indicated some room for improvement in defining healthy diets for the Malaysian population. However, pragmatically in this population with habitual inclusion of animal foods, positive messages for country-specific behavior modification should be directed to the home meal pattern. The HM pattern, with its low eating-out frequency (9 times/week), importantly also reflected food group variety. The negative message for population nutrition should address eating out behaviors related to the SSB pattern, as it was equivalent to dietary monotony based on high non-alcoholic beverage consumption and local fast food such as noodles. Future interventional research designed for this population should incorporate the new biomarkers of food intake and pattern adherence for targeted healthful behaviors.

## 5. Conclusions

Overall, we conclude that for our study population, who were essentially free of NCDs diagnosis, had a high cardiometabolic risk profile associated with the habitual consumption of the SSB pattern. The SSB pattern eating mode was reflective of a high intake of sugar-sweetened beverages alongside high fat-high carbohydrate food choices typically available when eating out, and this pattern amplified MetS risk.

## Figures and Tables

**Table 1:** Characteristics of Malaysia Lipid Study (MLS) study participants (*n* = 562).

Parameter	Value
Gender, *n* (%)	
Male	210 (37%)
Female	352 (63%)
Age, years	38.1 ± 11.4
Ethnicity, *n* (%)	
Malay	222 (40%)
Chinese	201 (36%)
Indian	139 (25%)
Education, *n* (%)	
No formal education	6 (1%)
Primary	35 (6%)
Secondary	180 (32%)
Tertiary, diploma/degree	336 (60%)
Others	3 (1%)
Physical activity level, *n* (%)	
Sedentary	118 (21%)
Moderately active	292 (52%)
Active	152 (27%)
Monthly household income, RM	4434 ± 3476
Cardiometabolic status	
BMI, kg/m^2^	24.8 ± 4.7
WC, cm	83.7 ± 12.8
TC, mmol/L	5.11 ± 0.90
LDL-C, mmol/L	3.10 ± 0.85
HDL-C, mmol/L	1.48 ± 0.39
Small LDL-C, nmol/L	472 ± 317
Large LDL-C, nmol/L	466 ± 211
Small HDL-C, nmol/L	14.93 ± 4.71
Large HDL-C, nmol/L	6.35 ± 3.25
TG, mmol/L	1.16 ± 0.60
TC: HDL-C	3.68 ± 1.14
FPG, mmol/L	5.10 ± 0.66
Insulin, uU/mL	6.09 ± 4.27
HOMA2-IR	0.82 ± 0.54
hsCRP, mg/L	2.84 ± 4.34
SBP, mmHg	123 ± 16
DBP, mmHg	75 ± 11

Abbreviations: BMI, body mass index; DBP, diastolic blood pressure; FPG, fasting plasma glucose; HDL, high density lipoprotein; HOMA2-IR, homeostatic model assessment of insulin resistance; hsCRP, high-sensitivity C-reactive protein; LDL-C, low density lipoprotein; SBP, systolic blood pressure; TC, total cholesterol; TC:HDL-C, total cholesterol high density lipoprotein ratio; TG, triglyceride; WC, waist circumference. Values are means ± SDs unless otherwise indicated

**Table 2:** Factor loadings for four dietary patterns in the MLS population (*n* = 562).

	Home Meal	Chinese Traditional	Plant Foods	Sugar Sweetened Beverages
White rice	**0.91**	0.02	0.00	−0.35
Sugar sweetened beverages	0.36	−0.24	−0.05	**0.90**
Non-starchy vegetables	0.32	0.06	0.29	−0.21
Fish and shellfish	0.26	−0.05	0.01	−0.12
Poultry	0.23	−0.05	0.00	0.02
Egg	0.14	−0.01	−0.04	0.08
Meat	0.11	0.04	0.00	0.01
Legumes	0.08	−0.12	0.03	0.00
Soybean products	0.05	0.05	0.10	−0.01
Unsweetened coffee and tea	0.01	0.33	−0.01	0.00
Chapati meal	−0.03	−0.12	−0.04	0.01
Wholemeal bread, biscuit and cereals	−0.03	−0.02	0.19	−0.05
Pau, dim sum and yong tau foo	−0.04	0.14	0.13	−0.03
Butter and margarine	−0.04	−0.06	0.15	0.04
Refined traditional meal	−0.05	−0.22	−0.02	0.09
Pizza, pasta and lasagna	−0.05	0.03	−0.06	0.14
Preserved vegetable	−0.07	0.18	0.01	−0.01
Milk, cheese and yogurt	−0.10	0.02	0.12	−0.08
Unsweetened fruit and vegetable juice	−0.15	0.04	0.02	−0.02
Noodle dishes	−0.16	**0.96**	−0.05	0.23
Fruit and dried fruit	−0.16	−0.01	**0.88**	−0.05
Fried rice, chicken rice and *nasi lemak*	−0.16	−0.23	−0.52	0.12
Brown rice and parboiled rice	−0.19	0.02	0.15	−0.06

Note: Dietary patterns extraction is by principal component analysis; positive and negative factor loadings equal to or more than 0.15 are indicated; numbers in bold indicate the maximum factor loading under each dietary pattern.

**Table 3:** Population characteristics as per dietary patterns.

	Home Meal			Chinese Traditional			Plant Foods			Sugar-Sweetened Beverages		
	T1 (*n* = 194)	T2 (*n* = 185)	T3 (*n* = 183)	T1 (*n* = 186)	T2 (*n* = 189)	T3 (*n* = 187)	T1 (*n* = 213)	T2 (*n* = 162)	T3 (*n* = 187)	T1 (*n* = 187)	T2 (*n* = 183)	T3 (*n* = 192)
Age, years	36.8 ± 11.5	37.5 ± 10.4	40.1 ± 12.0 ^a,b^	38.4 ± 10.9	37.2 ± 11.2	38.7 ± 12.0	36.7 ± 11.1	37.2 ± 10.8	40.5 ± 11.9 ^a^	37.9 ± 12.2	39.4 ± 11.7	37.0 ± 10.2
Sex, *n* (%)												
Male	63 (33)	56 (30)	91 (50) ^c^	77 (41)	68 (36)	65 (35)	97 (46)	56 (35)	57 (31) ^c^	45 (24)	60 (33)	105 (55) ^c^
Female	131 (68)	129 (70)	92 (50)	109 (59)	121 (64)	122 (65)	116 (55)	106 (65)	130 (70)	142 (76)	123 (67)	87 (45)
Ethnicity, *n* (%)												
Malay	69 (36)	73 (40)	80 (40)	84 (45)	72 (38)	66 (35) ^c^	94 (44)	66 (41)	62 (33) ^c^	58 (31)	75 (41)	89 (46) ^c^
Chinese	79 (41)	62 (34)	60 (33)	39 (21)	56 (30)	106 (57)	57 (27)	56 (35)	88 (47)	87 (47)	65 (36)	49 (26)
India	46 (24)	50 (27)	43 (24)	63 (34)	61 (32)	15 (8)	62 (29)	40 (25)	37 (20)	42 (23)	43 (24)	54 (28)
Education level, *n* (%)												
Secondary and lower	57 (30)	80 (43)	84 (46) ^c^	80 (43)	65 (34)	76 (41)	77 (36)	65 (40)	79 (42)	73 (39)	79 (43)	69 (36)
Tertiary and higher	136 (70)	105 (57)	98 (54)	105 (57)	124 (66)	110 (59)	135 (64)	96 (60)	108 (58)	113 (61)	103 (57)	123 (64)
Income, RM/month	4661 ± 3675	4514 ± 3419	4105 ± 3307	4416 ± 3489	4205 ± 3232	4688 ± 3701	4424 ± 3935	4361 ± 3069	4512 ± 3247	4326 ± 3488	4480 ± 3590	4490 ± 3370
Physical activity level, *n* (%)												
Sedentary	37 (19)	42 (23)	39 (21)	46 (25)	35 (19)	37 (20)	48 (23)	33 (21)	37 (20)	39 (21)	40 (22)	39 (20) ^c^
Moderately active	103 (53)	94 (51)	95 (52)	91 (49)	110 (58)	91 (49)	111 (52)	80 (49)	101 (54)	99 (53)	106 (58)	87 (45)
Active	54 (28)	49 (26)	49 (27)	49 (26)	44 (23)	59 (31)	54 (25)	49 (30)	49 (26)	49 (26)	37 (20)	66 (35)
Eating out, times/week	11 ± 8	10 ± 6	9 ± 7	9 ± 7	10 ± 8	11 ± 7 ^a,b^	10 ± 7	10 ± 7	9 ± 7 ^a,b^	8 ± 7	9 ± 6	12 ± 8 ^a,b^
Nutrient composition												
Total energy intake, kcal	1787 ± 365	1778 ± 383	1907 ± 453 ^a,b^	1763 ± 384	1841 ± 438	1865 ± 384 ^a,b^	1825 ± 411	1815 ± 415	1829 ± 390	1662 ± 301	1840 ± 417	1964 ± 426 ^a,b^
Carbohydrate, g	236.5 ± 56.5	241.1 ± 51.3	263.3 ± 62.1 ^a,b^	242.8 ± 56.1	249.7 ± 61.9	247.7 ± 55.6	244.1 ± 58.9	246.3 ± 58.0	250.2 ± 56.8	219.5 ± 43.6	249.5 ± 56.0	270.7 ± 60.8 ^a,b^
Protein, g	61.9 ± 17.0	61.5 ± 17.5	67.0 ± 20.9 ^a,b^	60.8 ± 17.5	61.9 ± 19.4	67.6 ± 18.4 ^a,b^	63.8 ± 19.0	62.6 ± 17.2	63.7 ± 19.5	58.1 ± 14.4	65.0 ± 20.2	67.1 ± 19.6 ^a,b^
Fat, g	65.3 ± 17.9	63.0 ± 19.8	64.7 ± 21.5	60.3 ± 18.3	65.9 ± 20.2	66.7 ± 20.1 ^a,b^	65.3 ± 19.3	64.1 ± 21.1	63.5 ± 19.1	60.6 ± 16.9	64.5 ± 21.2	67.8 ± 20.3 ^a,b^
Sodium, mg	3046 ± 1026	2950 ± 1064	3010 ± 1019	2671 ± 906	2928 ± 940	3409 ± 1115 ^a,b^	2983 ± 1038	3016 ± 1113	3013 ± 966	2925 ± 1063	3040 ± 1060	3043 ± 984
Food groups * consumed per day per subject												
White rice, g	67 (60)	160 (40)	281 (89) ^d^	183 (164)	157 (150)	133 (130) ^d^	143 (156)	160 (150)	157 (147)	160 (150)	160 (150)	143 (151)
Non-starchy vegetables, g	37 (61)	55 (72)	82 (87) ^d^	61 (88)	57 (79)	55 (83)	52 (76)	55 (75)	64 (90)	68 (91)	55 (73)	47 (77) ^d^
Fish and shellfish, g	10 (31)	20 (42)	26 (54) ^d^	22 (49)	18 (40)	17 (37)	20 (43)	18 (40)	20 (43)	15 (40)	25 (44)	18 (44)
Poultry, g	15 (50)	30 (60)	33 (77) ^d^	30 (67)	27 (58)	23 (60)	27 (69)	27 (57)	27 (60)	23 (50)	25 (63)	30 (74)
Egg, g	0 (17)	4 (17)	8 (23)	2 (23)	4 (23)	0 (15)	4 (18)	0 (15)	6 (25)	0 (15)	3 (19)	9 (31) ^d^
Legume, g	0 (14)	4 (20)	3 (22)	5 (24)	1 (19)	0 (13) ^d^	3 (19)	0 (16)	2 (25)	0 (18)	2 (22)	4 (19)
Fruit and dried fruit, g	13 (109)	25 (100)	40 (101)	24 (88)	25 (112)	32 (107)	0	32 (33)	153 (122) ^d^	44 (137)	28 (108)	11 (70) ^d^
Kuih, g	9 (54)	0 (50)	0(50)	7 (52)	4 (60)	0 (44)	0 (47)	13(54)	10(58)	0 (40)	17 (60)	19 (65) ^d^
Fried rice and *nasi lemak*, g	72 (132)	69 (138)	125 (143)	77 (167)	63 (125)	47 (115) ^d^	77 (155)	61 (131)	60 (112)	48 (111)	68 (149)	73 (143) ^d^
Noodle dishes, g	125 (243)	113 (227)	75 (190) ^d^	0	107 (61)	304 (178) ^d^	89 (205)	106 (244)	116 (212)	112 (207)	113 (250)	92 (209)
Refined traditional cereal, g	50 (96)	34 (83)	33 (77)	53 (98)	49 (98)	28 (62) ^d^	44 (89)	40 (84)	34 (78)	28 (68)	44 (90)	53 (85) ^d^
Sugary beverages, mL	338 (348)	249 (300)	342 (300)	375 (304)	341 (345)	316 (292)	380 (335)	350 (285)	300 (298)	167 (117)	339 (92)	605 (282) ^d^

Note: Values are means ± SDs or * median (interquartile range); ^a^ *p* < 0.05 for ANOVA test for comparison of continuous data between tertiles; ^b^ *p* < 0.05 between T3 vs. T1; ^c^ *p* < 0.05 for Chi-square test for comparison of categorical data between tertiles, ^d^ *p* < 0.05 for Kruskal-Wallis test. Glossary of terms: kuih = sweet traditional cakes, *nasi lemak*= rice cooked with coconut milk.

**Table 4:** Cardiometabolic risk markers of subjects across tertiles (T) of dietary patterns (*n* = 562).

	Home Meal			Chinese Traditional			Plant Foods			Sugar-Sweetened Beverages		
	Model 1	Model 2	Model 3	Model 1	Model 2	Model 3	Model 1	Model 2	Model 3	Model 1	Model 2	Model 3
BMI, kg/m^2^												
T1	24.2 ± 0.3	24.5 ± 0.3	24.6 ± 0.4	25.1 ± 0.3	25.3 ± 0.3	25.4 ± 0.4	25.0 ± 0.3	25.1 ± 0.3	25.4 ± 0.4	23.9 ± 0.3	24.0 ± 0.4	24.2 ± 0.4
T2	25.4 ± 0.4	25.6 ± 0.4	25.7 ± 0.4	25.0 ± 0.4	25.1 ± 0.3	25.3 ± 0.4	24.9 ± 0.4	25.1 ± 0.4	25.2 ± 0.4	25.1 ± 0.3	25.1 ± 0.4	25.2 ± 0.4
T3	24.7 ± 0.3	24.6 ± 0.3	24.9 ± 0.4	24.3 ± 0.3	24.4 ± 0.3	24.6 ± 0.4	24.5 ± 0.3	24.5 ± 0.4	24.6 ± 0.4	25.4 ± 0.3	25.5 ± 0.3	25.7 ± 0.4
*P*_trend_	0.040	0.026	0.012	0.20	0.18	0.21	0.49	0.31	0.23	0.003 **^†^**	0.007 **^†^**	0.012 **^†^**
WC, cm												
T1	81.5 ± 0.8	83.2 ± 0.9	83.4 ± 1.0	85.3 ± 0.9	85.9 ± 0.9	86.5 ± 1.0	84.5 ± 0.9	85.2 ± 0.8	85.8 ± 0.9	81.0 ± 1.0	82.8 ± 0.9	83.5 ± 1.0
T2	85.2 ± 1.0	86.9 ± 0.9	87.0 ± 1.0	84.2 ± 1.0	85.6 ± 0.9	85.9 ± 1.0	83.7 ± 1.0	85.1 ± 1.0	85.5 ± 1.0	83.8 ± 0.8	84.6 ± 0.9	84.9 ± 1.0
T3	84.6 ± 1.0	84.1 ± 0.9	85.0 ± 1.0	81.6 ± 0.8	82.5 ± 0.9	83.2 ± 1.0	82.8 ± 0.9	83.6 ± 0.9	84.0 ± 1.0	86.3 ± 1.0	86.3 ± 0.9	86.8 ± 1.0
*P*_trend_	0.010	0.007	0.016	0.014 **^†^**	0.011 **^†^**	0.021 **^†^**	0.44	0.33	0.31	<0.001 **^†^**	0.020 **^†^**	0.038 **^†^**
TC, mmol/L												
T1	5.13 ± 0.07	5.19 ± 0.06	5.17 ± 0.07	5.11 ± 0.06	5.11 ± 0.06	5.09 ± 0.07	5.07 ± 0.06	5.11 ± 0.06	5.08 ± 0.07	5.03 ± 0.07	5.04 ± 0.07	5.04 ± 0.07
T2	5.08 ± 0.06	5.12 ± 0.06	5.11 ± 0.07	5.05 ± 0.06	5.09 ± 0.06	5.07 ± 0.07	5.06 ± 0.06	5.10 ± 0.07	5.08 ± 0.07	5.13 ± 0.06	5.10 ± 0.06	5.09 ± 0.07
T3	5.11 ± 0.06	5.05 ± 0.06	5.02 ± 0.07	5.16 ± 0.07	5.16 ± 0.06	5.14 ± 0.07	5.19 ± 0.07	5.14 ± 0.06	5.15 ± 0.07	5.16 ± 0.06	5.19 ± 0.06	5.16 ± 0.07
*P*_trend_	0.86	0.32	0.31	0.48	0.70	0.69	0.33	0.91	0.66	0.30	0.24	0.47
LDL-C, mmol/L												
T1	3.09 ± 0.07	3.16 ± 0.06	3.15 ± 0.07	3.14 ± 0.06	3.16 ± 0.06	3.14 ± 0.06	3.10 ± 0.06	3.14 ± 0.06	3.11 ± 0.06	2.98 ± 0.07	3.03 ± 0.06	3.05 ± 0.07
T2	3.09 ± 0.06	3.15 ± 0.06	3.14 ± 0.07	3.03 ± 0.06	3.08 ± 0.06	3.07 ± 0.07	3.05 ± 0.06	3.10 ± 0.06	3.09 ± 0.07	3.15 ± 0.06	3.15 ± 0.06	3.14 ± 0.07
T3	3.10 ± 0.06	3.06 ± 0.06	3.04 ± 0.07	3.11 ± 0.07	3.13 ± 0.06	3.11 ± 0.07	3.13 ± 0.07	3.12 ± 0.06	3.13 ± 0.07	3.16 ± 0.06	3.18 ± 0.06	3.14 ± 0.07
*P*_trend_	0.99	0.41	0.43	0.43	0.68	0.67	0.64	0.89	0.90	0.07	0.20	0.45
HDL-C, mmol/L												
T1	1.53 ± 0.03	1.48 ± 0.03	1.47 ± 0.03	1.42 ± 0.03	1.40 ± 0.03	1.39 ± 0.03	1.44 ± 0.03	1.43 ± 0.03	1.42 ± 0.03	1.58 ± 0.03	1.51 ± 0.03	1.48 ± 0.03
T2	1.47 ± 0.03	1.41 ± 0.03	1.40 ± 0.03	1.50 ± 0.03	1.46 ± 0.03	1.46 ± 0.03	1.48 ± 0.03	1.44 ± 0.03	1.42 ± 0.03	1.44 ± 0.03	1.39 ± 0.03	1.40 ± 0.03
T3	1.45 ± 0.03	1.45 ± 0.03	1.44 ± 0.03	1.52 ± 0.03	1.48 ± 0.03	1.47 ± 0.03	1.53 ± 0.03	1.48 ± 0.03	1.47 ± 0.03	1.43 ± 0.03	1.44 ± 0.03	1.43 ± 0.03
*P*_trend_	0.10	0.22	0.26	0.05	0.12	0.10	0.08	0.28	0.37	<0.001	0.012	0.10
TG, mmol/L												
T1	1.11 ± 0.04	1.18 ± 0.04	1.19 ± 0.05	1.18 ± 0.04	1.20 ± 0.04	1.20 ± 0.04	1.16 ± 0.04	1.19 ± 0.04	1.19 ± 0.04	1.03 ± 0.04	1.10 ± 0.04	1.12 ± 0.05
T2	1.15 ± 0.04	1.22 ± 0.04	1.23 ± 0.05	1.12 ± 0.04	1.18 ± 0.04	1.17 ± 0.05	1.17 ± 0.05	1.23 ± 0.05	1.23 ± 0.05	1.19 ± 0.05	1.22 ± 0.04	1.20 ± 0.05
T3	1.21 ± 0.05	1.19 ± 0.04	1.18 ± 0.05	1.17 ± 0.04	1.21 ± 0.04	1.22 ± 0.05	1.14 ± 0.04	1.17 ± 0.04	1.19 ± 0.05	1.25 ± 0.04	1.25 ± 0.04	1.27 ± 0.04
*P*_trend_	0.24	0.82	0.68	0.60	0.85	0.71	0.94	0.63	0.77	0.001 **^†^**	0.026 **^†^**	0.053
TC:HDL-C												
T1	3.62 ± 0.09	3.79 ± 0.08	3.80 ± 0.09	3.83 ± 0.08	3.88 ± 0.08	3.90 ± 0.08	3.75 ± 0.08	3.82 ± 0.07	3.80 ± 0.08	3.38 ± 0.08	3.56 ± 0.08	3.62 ± 0.09
T2	3.68 ± 0.08	3.85 ± 0.08	3.86 ± 0.09	3.62 ± 0.09	3.74 ± 0.08	3.74 ± 0.09	3.69 ± 0.10	3.83 ± 0.08	3.86 ± 0.09	3.81 ± 0.08	3.90 ± 0.08	3.87 ± 0.09
T3	3.75 ± 0.08	3.71 ± 0.08	3.71 ± 0.09	3.61 ± 0.08	3.70 ± 0.08	3.72 ± 0.09	3.60 ± 0.08	3.68 ± 0.08	3.72 ± 0.09	3.86 ± 0.08	3.85 ± 0.08	3.87 ± 0.08
*P*_trend_	0.53	0.43	0.42	0.11	0.22	0.22	0.40	0.34	0.50	<0.001 **^†^**	0.004 **^†^**	0.039
Small LDL, nmol/L												
T1	449.7 ± 23.9	488.6 ± 22.0	449.7 ± 24.9	488.8 ± 21.3	501.3 ± 22.1	505.6 ± 23.5	472.0 ± 21.6	488.6 ± 20.6	482.3 ± 22.6	390.7 ± 21.7	432.8 ± 22.8	449.7 ± 24.9
T2	480.8 ± 23.7	518.7 ± 22.6	534.0 ± 24.6	456.7 ± 25.5	485.6 ± 22.2	480.5 ± 25.2	478.2 ± 24.7	510.1 ± 23.9	515.2 ± 25.5	526.4 ± 23.5	545.7 ± 22.4	534.0 ± 24.6
T3	486.2 ± 21.8	474.1 ± 22.2	503.2 ± 23.6	470.1 ± 22.6	491.9 ± 22.3	499.4 ± 24.1	466.0 ± 23.7	483.3 ± 22.7	496.2 ± 25.2	498.3 ± 23.3	497.8 ± 21.4	503.2 ± 23.6
*P*_trend_	0.48	0.36	0.028	0.62	0.88	0.71	0.94	0.68	0.58	<0.001	0.001	0.028
Large LDL, nmol/L												
T1	472.4 ± 16.1	467.6 ± 15.3	451.9 ± 17.9	448.9 ± 14.4	444.0 ± 15.5	433.6 ± 16.8	449.9 ± 13.8	451.8 ± 14.4	441.3 ± 16.0	481.1 ± 14.8	470.0 ± 16.2	448.4 ± 18.1
T2	446.8 ± 15.4	438.5 ± 15.8	446.1 ± 17.7	465.0 ± 16.6	461.4 ± 15.4	452.6 ± 17.8	468.8 ± 15.7	464.6 ± 16.9	455.2 ± 18.3	457.8 ± 15.9	445.7 ± 15.9	447.5 ± 17.7
T3	477.5 ± 15.0	471.6 ± 15.6	447.7 ± 16.7	482.6 ± 15.5	474.1 ± 15.6	460.8 ± 17.1	480.8 ± 17.2	465.3 ± 15.8	451.8 ± 17.8	458.1 ± 15.9	463.1 ± 15.1	450.9 ± 16.9
*P*_trend_	0.33	0.26	0.97	0.31	0.38	0.46	0.34	0.78	0.80	0.48	0.52	0.99
Small HDL, nmol/L												
T1	14.6 ± 0.4	15.2 ± 0.3	15.7 ± 0.4	15.4 ± 0.3	15.5 ± 0.3	15.8 ± 0.3	14.7 ± 0.3	15.0 ± 0.3	15.4 ± 0.3	14.4 ± 0.4	15.1 ± 0.3	15.5 ± 0.4
T2	14.7 ± 0.3	15.2 ± 0.3	15.6 ± 0.4	14.1 ± 0.4	14.5 ± 0.3	14.9 ± 0.4	15.2 ± 0.4	15.7 ± 0.4	15.9 ± 0.4	15.3 ± 0.4	15.5 ± 0.3	16.0 ± 0.4
T3	15.5 ± 0.3	15.3 ± 0.3	15.6 ± 0.4	15.4 ± 0.3	15.7 ± 0.3	16.0 ± 0.4	14.9 ± 0.4	15.1 ± 0.3	15.7 ± 0.4	15.1 ± 0.3	15.1 ± 0.3	15.4 ± 0.4
*P*_trend_	0.10	0.97	0.99	0.009	0.019	0.024	0.63	0.28	0.48	0.20	0.57	0.45
Large HDL, nmol/L												
T1	6.81 ± 0.25	6.24 ± 0.21	6.08 ± 0.24	5.84 ± 0.23	5.60 ± 0.21	5.43 ± 0.23	5.99 ± 0.22	5.81 ± 0.20	5.73 ± 0.22	7.29 ± 0.24	6.54 ± 0.22	6.30 ± 0.25
T2	6.24 ± 0.23	5.63 ± 0.22	5.55 ± 0.24	6.64 ± 0.24	6.20 ± 0.21	6.13 ± 0.25	6.38 ± 0.27	5.89 ± 0.23	5.77 ± 0.25	6.00 ± 0.23	5.53 ± 0.22	5.51 ± 0.24
T3	5.97 ± 0.23	6.02 ± 0.22	5.92 ± 0.25	6.56 ± 0.24	6.13 ± 0.22	6.03 ± 0.24	6.74 ± 0.23	6.24 ± 0.22	6.05 ± 0.25	5.78 ± 0.24	5.88 ± 0.21	5.73 ± 0.23
*P*_trend_	0.038	0.12	0.21	0.033	0.09	0.05	0.07	0.32	0.53	<0.001 **^†^**	0.003	0.036
FPG, mmol/L												
T1	5.03 ± 0.04	5.09 ± 0.05	5.08 ± 0.05	5.15 ± 0.05	5.17 ± 0.05	5.16 ± 0.05	5.11 ± 0.04	5.14 ± 0.04	5.13 ± 0.05	5.06 ± 0.04	5.12 ± 0.05	5.11 ± 0.05
T2	5.09 ± 0.06	5.16 ± 0.05	5.14 ± 0.05	5.07 ± 0.06	5.12 ± 0.05	5.11 ± 0.05	5.10 ± 0.05	5.16 ± 0.05	5.13 ± 0.05	5.05 ± 0.04	5.07 ± 0.05	5.05 ± 0.05
T3	5.18 ± 0.04	5.15 ± 0.05	5.13 ± 0.05	5.08 ± 0.04	5.11 ± 0.05	5.09 ± 0.05	5.08 ± 0.06	5.10 ± 0.05	5.09 ± 0.05	5.19 ± 0.06	5.19 ± 0.05	5.19 ± 0.05
*P*_trend_	0.07	0.54	0.62	0.46	0.64	0.56	0.94	0.67	0.79	0.07	0.18	0.09
Insulin, uU/mL												
T1	5.88 ± 0.29	6.09 ± 0.31	6.21 ± 0.36	6.18 ± 0.28	6.27 ± 0.31	6.38 ± 0.34	6.54 ± 0.32	6.60 ± 0.29	6.72 ± 0.33	5.22 ± 0.25	5.44 ± 0.32	5.59 ± 0.36
T2	6.26 ± 0.34	6.49 ± 0.32	6.58 ± 0.35	6.21 ± 0.34	6.37 ± 0.31	6.50 ± 0.37	6.12 ± 0.32	6.29 ± 0.34	6.40 ± 0.37	6.45 ± 0.35	6.58 ± 0.32	6.65 ± 0.36
T3	6.13 ± 0.31	6.11 ± 0.32	6.27 ± 0.36	5.88 ± 0.31	6.03 ± 0.32	6.23 ± 0.35	5.55 ± 0.29	5.70 ± 0.32	5.85 ± 0.36	6.59 ± 0.32	6.56 ± 0.31	6.77 ± 0.35
*P*_trend_	0.67	0.60	0.68	0.71	0.73	0.84	0.07	0.11	0.15	0.003 **^†^**	0.014 **^†^**	0.022 **^†^**
HOMA2-IR												
T1	0.79 ± 0.04	0.82 ± 0.04	0.83 ± 0.05	0.83 ± 0.03	0.85 ± 0.04	0.86 ± 0.04	0.88 ± 0.04	0.89 ± 0.04	0.90 ± 0.04	0.71 ± 0.03	0.74 ± 0.04	0.76 ± 0.05
T2	0.85 ± 0.04	0.88 ± 0.04	0.89 ± 0.04	0.84 ± 0.04	0.86 ± 0.04	0.88 ± 0.05	0.83 ± 0.04	0.85 ± 0.04	0.86 ± 0.05	0.87 ± 0.04	0.89 ± 0.04	0.89 ± 0.05
T3	0.83 ± 0.04	0.83 ± 0.04	0.84 ± 0.05	0.79 ± 0.04	0.82 ± 0.04	0.84 ± 0.04	0.76 ± 0.04	0.78 ± 0.04	0.79 ± 0.05	0.89 ± 0.04	0.88 ± 0.04	0.91 ± 0.04
*P*_trend_	0.57	0.51	0.59	0.66	0.68	0.79	0.08	0.12	0.16	0.002 **^†^**	0.014 **^†^**	0.023 **^†^**
hsCRP, mg/L												
T1	2.31 ± 0.28	2.24 ± 0.32	2.18 ± 0.36	3.53 ± 0.38	3.46 ± 0.32	3.38 ± 0.35	2.93 ± 0.28	2.95 ± 0.30	2.91 ± 0.34	2.21 ± 0.29	2.01 ± 0.33	2.03 ± 0.37
T2	3.29 ± 0.31	3.18 ± 0.33	3.08 ± 0.35	2.57 ± 0.23	2.50 ± 0.32	2.44 ± 0.37	2.91 ± 0.36	2.84 ± 0.35	2.82 ± 0.38	3.46 ± 0.36	3.27 ± 0.33	3.20 ± 0.37
T3	2.96 ± 0.35	2.89 ± 0.32	2.91 ± 0.37	2.44 ± 0.32	2.31 ± 0.32	2.31 ± 0.36	2.69 ± 0.32	2.46 ± 0.33	2.46 ± 0.37	2.88 ± 0.30	2.95 ± 0.31	2.95 ± 0.35
*P*_trend_	0.08	0.09	0.12	0.029 **^†^**	0.022 **^†^**	0.043 **^†^**	0.84	0.52	0.58	0.022	0.015	0.035
SBP, mmHg												
T1	121.3 ± 1.1	123.4 ± 1.1	123.0 ± 1.2	122.9 ± 1.1	123.4 ± 1.1	122.9 ± 1.1	123.1 ± 1.1	124.2 ± 1.0	123.5 ± 1.1	119.5 ± 1.0	121.6 ± 1.1	121.1 ± 1.2
T2	121.8 ± 1.1	123.7 ± 1.1	122.9 ± 1.1	122.3 ± 1.2	124.0 ± 1.1	122.7 ± 1.2	122.3 ± 1.2	124.0 ± 1.1	123.3 ± 1.2	124.8 ± 1.3	125.4 ± 1.1	124.3 ± 1.2
T3	125.7 ± 1.3	124.6 ± 1.1	123.3 ± 1.2	123.4 ± 1.2	124.3 ± 1.1	123.5 ± 1.2	123.2 ± 1.2	123.4 ± 1.1	122.3 ± 1.2	124.3 ± 1.1	124.6 ± 1.0	123.6 ± 1.1
*P*_trend_	0.015 **^†^**	0.72	0.95	0.81	0.83	0.86	0.85	0.85	0.67	0.002	0.029	0.08
DBP, mmHg												
T1	74.7 ± 0.7	75.9 ± 0.7	75.8 ± 0.8	75.0 ± 0.8	75.3 ± 0.7	75.3 ± 0.8	75.1 ± 0.8	75.7 ± 0.7	75.5 ± 0.8	72.9 ± 0.7	74.1 ± 0.8	74.1 ± 0.9
T2	74.9 ± 0.8	76.0 ± 0.8	75.7 ± 0.8	75.3 ± 0.8	76.2 ± 0.7	75.8 ± 0.9	74.8 ± 0.8	75.8 ± 0.8	75.6 ± 0.9	76.3 ± 0.8	76.7 ± 0.8	76.3 ± 0.8
T3	75.6 ± 0.8	75.1 ± 0.8	74.7 ± 0.9	74.8 ± 0.7	75.4 ± 0.8	75.2 ± 0.8	75.1 ± 0.8	75.4 ± 0.8	75.2 ± 0.9	76.0 ± 0.8	76.2 ± 0.7	75.8 ± 0.8
*P*_trend_	0.68	0.64	0.55	0.89	0.62	0.85	0.95	0.94	0.90	0.003	0.035	0.10

Abbreviations: BMI, body mass index; DBP, diastolic blood pressure; FPG, fasting plasma glucose; HDL, high density lipoprotein; HOMA2-IR, homeostatic model assessment of insulin resistance; hsCRP, high-sensitivity C-reactive protein; LDL-C, low density lipoprotein; SBP, systolic blood pressure; T1, tertile 1; T2, tertile 2; T3, tertile 3; TC, total cholesterol; TC:HDL-C, total cholesterol high density lipoprotein ratio; TG, triglyceride; WC, waist circumference. Data are expressed as mean ± SE; Model 1 is unadjusted. Model 2 is adjusted for age and gender; Model 3 is adjusted for Model 2 and education level, income, and physical activity level; General Linear Model tested comparisons between tertiles; Bonferroni correction for comparisons between T3 and T1; Values for *P*_trend_ < 0.05 are significant; **^†^** Significance between T3 vs. T1.

## Supplementary Materials

The following are available online at https://www.mdpi.com/2072-6643/12/7/2080/s1, Table S1: Adjusted odds ratio (AOR) for tertile 3 (T3) vs. tertile 1 (T1) DP comparisons for selected variables.
