# Supplementary material for: Associations of Eating Mode Defined by Dietary Patterns with Cardiometabolic Risk Factors in the Malaysia Lipid Study Population
Source: Nutrients. 2020 Jul 14;12(7):2080. doi: 10.3390/nu12072080 (PMC7400910; doi:10.3390/nu12072080)
Supplement: Supplementary file 1 [file nutrients-12-02080-s001.pdf]

Table S1: Adjusted odds ratio (AOR) for tertile 3 (T3) *vs* tertile 1 (T1) DP comparisons for selected variables

Variables	Home Meal*		Chinese Traditional*		Plant Food*		Sugar Sweetened Beverages*	
	AOR (95% CI)	*P*	AOR (95% CI)	*P*	AOR (95% CI)	*P*	AOR (95% CI)	*P*
BMI ≥ 25 kg/m²	0.83 (0.53,1.31)	0.424	0.82 (0.53,1.26)	0.358	0.70 (0.45,1.07)	0.101	2.01 (1.28,3.17)	0.003
WC ≥ 90 cm (men) or ≥ 80 cm (women)	1.03 (0.65,1.63)	0.906	0.66 (0.42,1.03)	0.066	0.80 (0.51,1.24)	0.311	1.81 (1.14,2.87)	0.013
TC ≥ 5.18 mmol/l	0.73 (0.46,1.15)	0.172	1.10 (0.71,1.71)	0.660	1.10 (0.72,1.69)	0.657	1.14 (0.72,1.78)	0.579
LDL-C ≥ 4.9 mmol/l	0.23 (0.05,1.15)	0.071	1.57 (0.36,6.89)	0.551	0.86 (0.20,3.70)	0.839	2.23 (0.56,8.88)	0.603
Small LDL-C particle ≥ 600 nmol/l	1.06 (0.65,1.72)	0.830	1.04 (0.65,1.67)	0.866	1.12 (0.69,1.80)	0.652	1.69 (1.02,2.79)	0.043
Large LDL-C < 450 mmol/l	0.85 (0.55,1.32)	0.476	0.84 (0.55,1.29)	0.436	0.82 (0.54,1.25)	0.355	1.22 (0.79,1.88)	0.380
HDL-C < 1.0 mmol/l (men) or < 1.3 mmol/l (women)	1.19 (0.63,2.23)	0.600	0.76 (0.42,1.38)	0.369	0.71 (0.38,1.30)	0.264	1.64 (0.84,3.18)	0.144
TG ≥ 1.7 mmol/l	0.73 (0.38,1.38)	0.327	0.92 (0.50,1.70)	0.794	0.80 (0.44,1.47)	0.477	1.94 (1.00,3.77)	0.050
TC:HDL-C > 4.5	0.69 (0.40,1.19)	0.184	0.67 (0.39,1.14)	0.141	0.82 (0.48,1.38)	0.449	1.58 (0.89,2.80)	0.119
FPG ≥ 5.6 mmol/l	1.11 (0.61,2.00)	0.736	0.99 (0.55,1.78)	0.963	0.95 (0.53,1.69)	0.850	1.46 (0.79,2.68)	0.224
HOMA2-IR > 1.4	0.99 (0.51, 1.92)	0.972	1.31 (0.67, 2.58)	0.431	0.47 (0.25, 0.91)	0.025	2.63 (1.25, 5.57)	0.011
hsCRP ≥ 1.0 mg/L	1.57 (1.00,2.45)	0.049	0.44 (0.28,0.70)	<0.001	0.70 (0.46,1.08)	0.111	2.21 (1.40,3.50)	0.001
SBP > 130 mmHg	1.25 (0.74,2.12)	0.396	1.08 (0.64,1.82)	0.774	1.10 (0.66,1.82)	0.717	1.68 (0.96,2.93)	0.069
DBP > 85 mmHg	0.72 (0.40,1.32)	0.289	1.25 (0.69,2.27)	0.467	0.90 (0.51,1.60)	0.728	1.42 (0.74,2.74)	0.297
MetS (Yes)	1.23 (0.68,2.23)	0.494	0.88 (0.49,1.57)	0.657	0.70 (0.40,1.23)	0.218	2.78 (1.49,5.22)	0.001

Footnotes: Data are expressed as odds ratio (95% CI) of T3 against odd ratio of T1 set as 1.0; †*Multiple logistic regression* test for comparison between tertiles (T3 *vs* T1) adjusted for age, gender, physical activity level, income, and education level. Abbreviations: AOR, adjusted odds ratio; BMI, body mass index; CI, confidence interval; DBP, diastolic blood pressure; FPG, fasting plasma glucose; HDL, high density lipoprotein; HOMA2-IR, homeostatic model assessment of insulin

resistance; hsCRP, high-sensitivity C-reactive protein; LDL-C, low density lipoprotein; SBP, systolic blood pressure; TC, total cholesterol; TC:HDL-C, total cholesterol high density lipoprotein ratio; TG, triglyceride; WC, waist circumference
